# Pili canaliculi caused by cetuximab – A three-dimensional ultrastructural analysis^☆^

**DOI:** 10.1016/j.abd.2023.04.010

**Published:** 2024-03-04

**Authors:** Hiram Larangeira de Almeida, Debora Sarzi Sartori, Felipe Yusuke Sato Shinzato, Samuel da Silva Julião, Sílvia Saueressig

**Affiliations:** a Postgraduation in Health and Behavior, Universidade Católica de Pelotas, Pelotas, RS, Brazil; b Department of Dermatology, Universidade Federal de Pelotas, Pelotas, RS, Brazil; c Universidade Federal de Pelotas, Pelotas, RS, Brazil

*Dear Editor,*

Monoclonal antibodies (mAb) have, since the end of the 1990s, been increasingly used in antitumor therapies, as part of antineoplastic treatments called targeted therapy. Since they have the advantage of being directed specifically and mainly to the lesion and its microenvironment, their use reduces damage to healthy cells and most adverse effects of non-specific therapies.^1^

Due to binding to the extracellular portion of epithelial growth factor receptors (EGFR) and interrupting the coupling of their usual ligands, anti-EGFR monoclonal antibodies prevent the cascade reactions triggered by their activation. As a rule, activation of these receptors, which are known to be part of a family comprising four distinct members, but which share common structural elements, culminates in cell proliferation, angiogenesis, inhibition of apoptosis, and metastasis. For this reason, EGFR blockade has been used as a targeted therapy for several neoplasms that produce their overexpression, which is, in itself, considered a criterion for worse prognosis.^2^, ^3^

EGFR inhibition also affects the proliferation of non-neoplastic cells, due to the natural presence of these receptors in keratinocytes, sebaceous glands and hair follicles. Therefore, this therapeutic modality has the potential to trigger adverse cutaneous effects that, although generally well tolerated and self-limited, when severe can restrict the use of these medications.^4^

Among the most common dermatological reactions is the acneiform rash that appears on the trunk and face, without the presence of comedones. Nail and hair involvement and the appearance of telangiectasias can also be part of the condition; paronychia, pyogenic granuloma, alopecia, eyelash trichomegaly, and facial hypertrichosis can also be observed. Studies demonstrate that lengthening and straightening of eyelashes, associated with changes in hair texture, can be present with ultra-structural and subclinical changes. Another less common effect is the appearance of melanocytic nevi and angioedema.^5^

The effects of three drugs that act by inhibiting EGFR ‒ panitumumab, erlotinib and gefitinib ‒ have already been evaluated using scanning electron microscopy, and hair grooving and twisted hair shafts have been observed. Through these investigations, changes were evidenced that generated clinical or subclinical variants of the phenotype described as *pili canaliculi*, characterized clinically by curly or wavy hair.^6^

Cetuximab is a monoclonal antibody that inhibits EGFR and, in combination with chemotherapy, is approved as a first-line treatment for metastatic colorectal cancer with expression of the aforementioned receptor, and even as isolated therapy in patients intolerant to chemotherapy.

Even though they have action mechanisms that are similar to those of drugs whose effects on hair have already been observed, it is speculated whether the structural differences between cetuximab and these other drugs may result in different effects. In this context, the present study aims to observe, using scanning electron microscopy, the three-dimensional ultrastructure of the eyelashes and hair shafts of a patient receiving this therapy.

A 64-year-old female patient who presented with colon carcinoma and liver metastases was treated with chemotherapy (FOLFIRI protocol: fluorouracil + leucovorin + irinotecan) and cetuximab for 12 months. She was referred for the treatment of acneiform rash on the trunk and face, which responded to treatment with oral tetracycline (500 mg twice a day for ten days). She reported changes in hair waviness (Fig. 1), which became frizzy after the start of the oncological therapy. The eyelashes changed their curvature and became elongated (Fig. 1).

**Figure 1:**
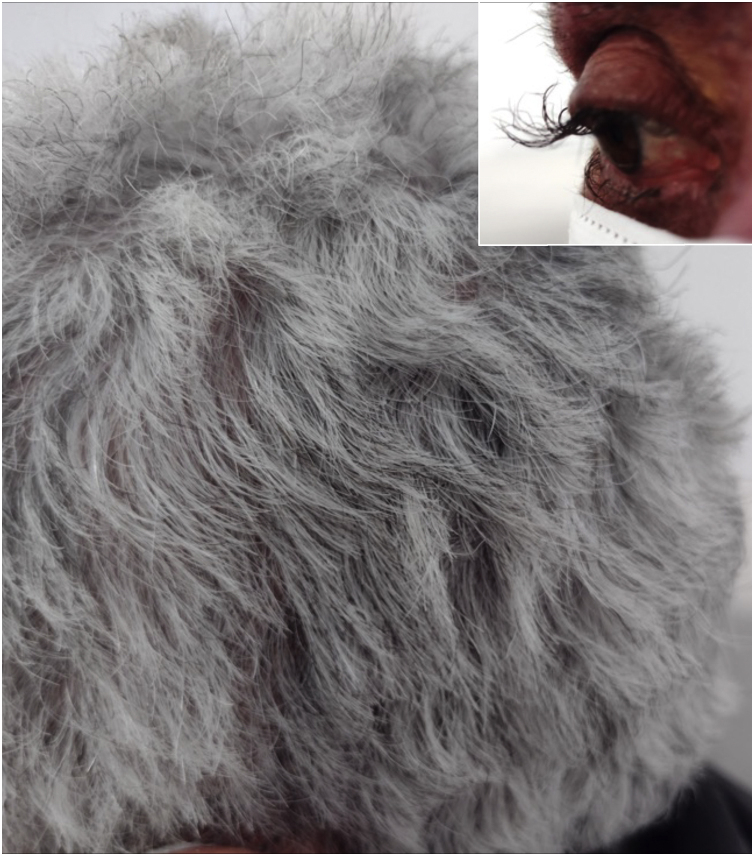
Clinical appearance with curly hair, elongated eyelashes in the insert.

Hair shafts and eyelash samples were collected and examined *in natura* using scanning electron microscopy.

Hair shafts showed on low power, longitudinal channels and discrete twists (Fig. 2). On higher magnifications, the channels become quite evident (Fig. 3). The examination of the eyelashes showed more pronounced changes, with well-developed, double (Fig. 4a) or single (Fig. 4b) channels.

**Figure 2:**
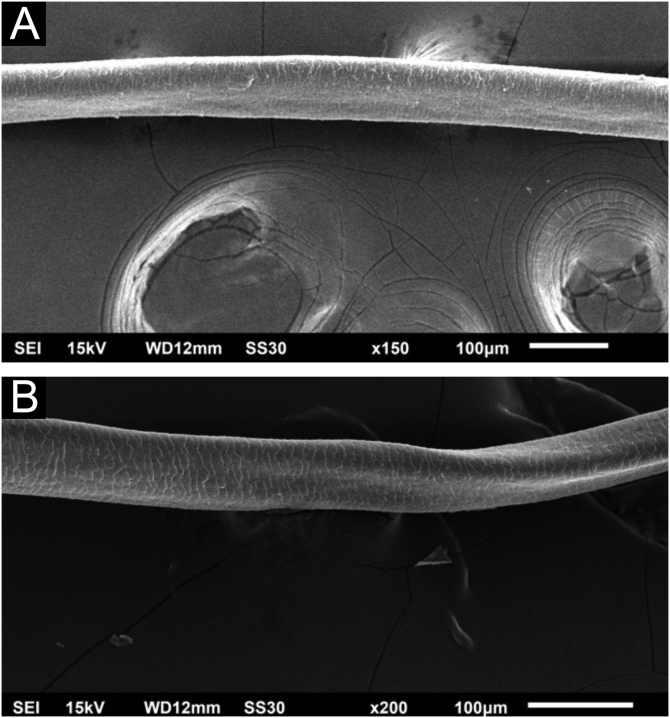
Scanning electron microscopy – (A) Hair shaft with longitudinal channel (×150). (B) Hair shaft with longitudinal channel and discrete twisting (×200).

**Figure 3:**
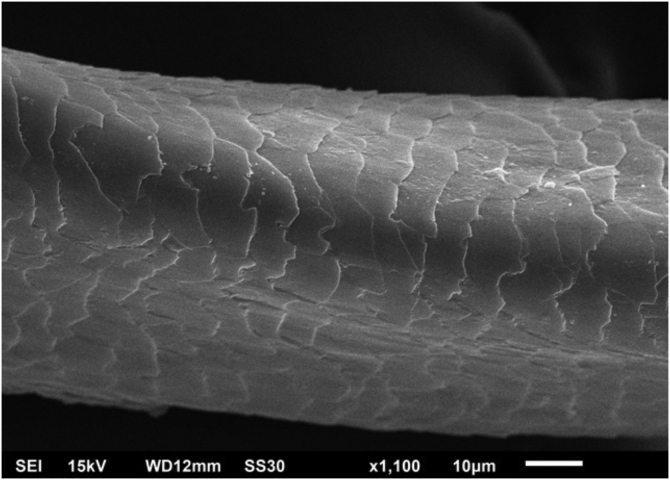
Scanning electron microscopy – high magnification detail of channel and twisting (×1.100).

**Figure 4:**
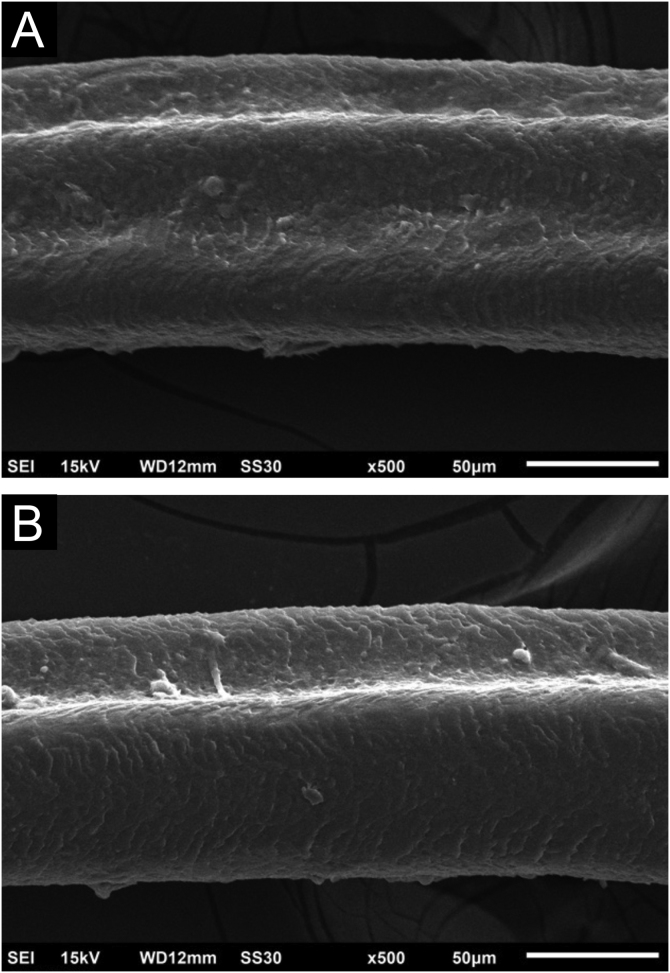
Scanning electron microscopy – (A) Eyelash with two channels (×500); (B) Eyelash with clearly visible single channel (×500).

These findings show that the change caused by cetuximab causes channels in the shafts, they may modify the elasticity of hairs shafts, altering their curvature or making them frizzy. These changes overlap with those caused by panitumumab^6^ and oral EGF inhibitors and are similar to the genetic forms of *pili canaliculi*, which are within the spectrum of the uncombable hair syndrome. The twisting was discreet, not characterizing *pili torti*, as they would have to rotate 180 degrees.^7^

With both cetuximab and panitumumab,^6^ eyelashes show ultra-structurally more prominent channels, and the authors presume that this may be due to their slower or shorter anagen phase.

Other medications used concomitantly, following the protocol for colon cancer metastatic disease, should not be the cause of the hair changes reported herein, as these are characteristic of EGFR inhibitors.

## Financial support

None declared.

## Authors' contributions

Hiram Larangeira de Almeida Jr.: Approval of the final version of the manuscript; design and planning of the study; drafting and editing of the manuscript; collection, analysis, and interpretation of data; effective participation in research orientation; intellectual participation in the propaedeutic and/or therapeutic conduct of the studied cases; critical review of the literature; critical review of the manuscript.

Debora Sarzi Sartori: Approval of the final version of the manuscript; design and planning of the study; drafting and editing of the manuscript; collection, analysis, and interpretation of data; intellectual participation in the propaedeutic and/or therapeutic conduct of the studied cases; critical review of the literature; critical review of the manuscript;

Felipe Yusuke Sato Shinzato: Approval of the final version of the manuscript; design and planning of the study; drafting and editing of the manuscript; collection, analysis, and interpretation of data; intellectual participation in the propaedeutic and/or therapeutic conduct of the studied cases; critical review of the literature; critical review of the manuscript.

Samuel da Silva Julião: Approval of the final version of the manuscript; design and planning of the study; drafting and editing of the manuscript; collection, analysis, and interpretation of data; intellectual participation in the propaedeutic and/or therapeutic conduct of the studied cases; critical review of the literature; critical review of the manuscript.

Sílvia Saueressig: Approval of the final version of the manuscript; design and planning of the study; drafting and editing of the manuscript; collection, analysis, and interpretation of data; intellectual participation in the propaedeutic and/or therapeutic conduct of the studied cases; critical review of the literature; critical review of the manuscript.

## Conflicts of interest

None declared.
